# Supplementary material for: Molecular mechanisms of splenectomy-induced hepatocyte proliferation
Source: PLoS One. 2020 Jun 12;15(6):e0233767. doi: 10.1371/journal.pone.0233767 (PMC7292681; doi:10.1371/journal.pone.0233767)

Ponceau S staining of liver/spleen samples

Image acquisition method: Photocamera

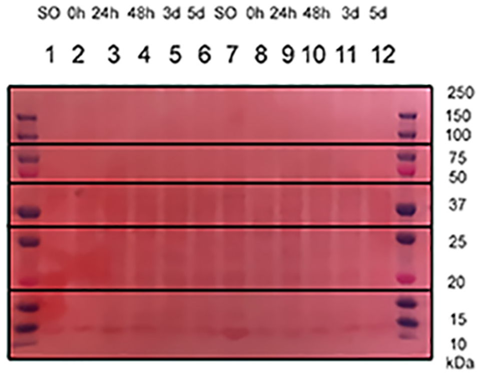

SO - sham-operated control

Figure 2

Image acquisition method: chemiluminescence detection

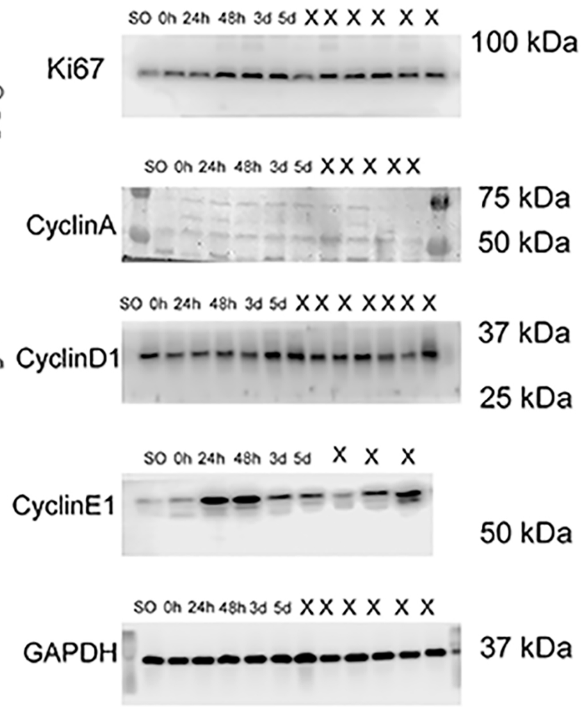

Figure 6

Image acquisition method: chemiluminescence detection

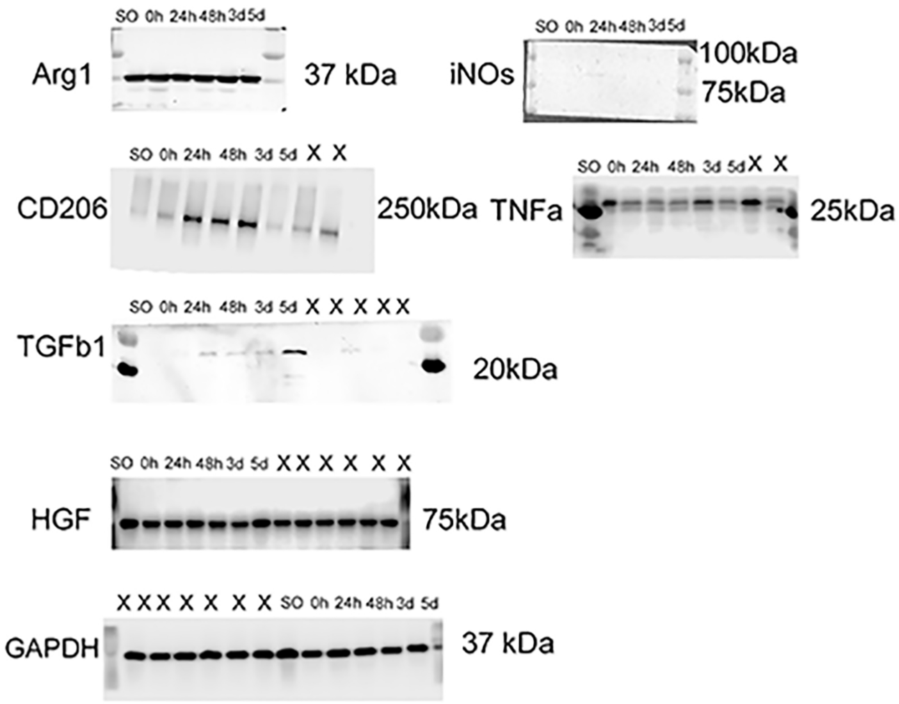

Figure 4

Image acquisition method: chemiluminescence detection

Ponceau S staining of liver/spleen samples

Image acquisition method: Photocamera

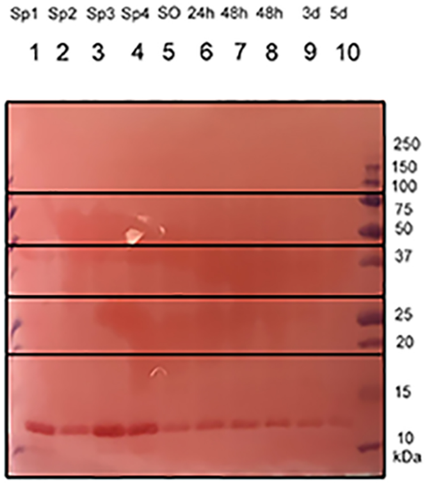

Sp - Spleen Sample; SO - sham-operated control

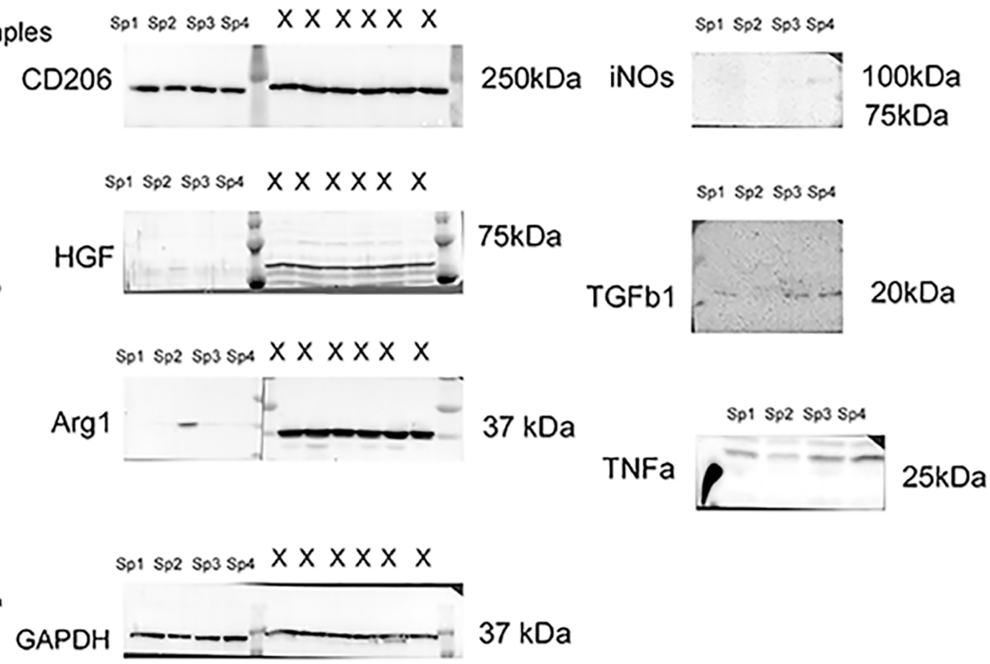

Supplement: S1 Fig — Staining of total proteins with Ponceau S and cutting of the blot for immunostaining are presented. After visualization of the proteins with Ponceau S, the membranes were cut as indicated with a dotted line. (PDF) [file pone.0233767.s001.pdf]
